# Endothelin-1 Mediates the Systemic and Renal Hemodynamic Effects of GPR81 Activation

**DOI:** 10.1161/HYPERTENSIONAHA.119.14308

**Published:** 2020-03-23

**Authors:** Natalie K. Jones, Kevin Stewart, Alicja Czopek, Robert I. Menzies, Adrian Thomson, Carmel M. Moran, Carolynn Cairns, Bryan R. Conway, Laura Denby, Dawn E.W. Livingstone, John Wiseman, Patrick W. Hadoke, David J. Webb, Neeraj Dhaun, James W. Dear, John J. Mullins, Matthew A. Bailey

**Affiliations:** 1 From the University/British Heart Foundation Centre for Cardiovascular Science, The University of Edinburgh, Scotland, United Kingdom (N.K.J., K.S., A.C., R.I.M., A.T., C.M.M., C.C., B.R.C., L.D., D.E.W.L., P.W.H., D.J.W., N.D., J.W.D., J.J.M., M.A.B.); 2 Discovery Sciences, IMED Biotech Unit, AstraZeneca R&D Gothenburg, Sweden (J.W.).

**Keywords:** blood pressure, hypoxia, ischemia-reperfusion-injury, pericytes, renal blood flow

## Abstract

GPR81 (G-protein-coupled receptor 81) is highly expressed in adipocytes, and activation by the endogenous ligand lactate inhibits lipolysis. GPR81 is also expressed in the heart, liver, and kidney, but roles in nonadipose tissues are poorly defined. GPR81 agonists, developed to improve blood lipid profile, might also provide insights into GPR81 physiology. Here, we assessed the blood pressure and renal hemodynamic responses to the GPR81 agonist, AZ′5538. In male wild-type mice, intravenous AZ′5538 infusion caused a rapid and sustained increase in systolic and diastolic blood pressure. Renal artery blood flow, intrarenal tissue perfusion, and glomerular filtration rate were all significantly reduced. AZ′5538 had no effect on blood pressure or renal hemodynamics in *Gpr81*^−/−^ mice. *Gpr81* mRNA was expressed in renal artery vascular smooth muscle, in the afferent arteriole, in glomerular and medullary perivascular cells, and in pericyte-like cells isolated from kidney. Intravenous AZ′5538 increased plasma ET-1 (endothelin 1), and pretreatment with BQ123 (endothelin-A receptor antagonist) prevented the pressor effects of GPR81 activation, whereas BQ788 (endothelin-B receptor antagonist) did not. Renal ischemia-reperfusion injury, which increases renal extracellular lactate, increased the renal expression of genes encoding ET-1, KIM-1 (Kidney Injury Molecule 1), collagen type 1-α1, TNF-α (tumor necrosis factor-α), and F4/80 in wild-type mice but not in *Gpr81*^−/−^ mice. In summary, activation of GPR81 in vascular smooth muscle and perivascular cells regulates renal hemodynamics, mediated by release of the potent vasoconstrictor ET-1. This suggests that lactate may be a paracrine regulator of renal blood flow, particularly relevant when extracellular lactate is high as occurs during ischemic renal disease.

## Abstract 2

Supplemental Digital Content is available in the text.

GPR81 (G-protein-coupled receptor 81), GPR109a, and GPR109b form the hydroxycarboxylic acid receptor subfamily.^1^ Encoded by the *HCAR1* gene, GPR81 is predominantly expressed in brown and white adipose tissue.^2^ L-lactate is the endogenous ligand with an EC_50_ of ≈5 mmol/L.^3^ Lactate, formed from pyruvate during anaerobic glycolysis, has a physiological plasma concentration of 0.5 to 2 mmol/L, rising from 10 to 30 mmol/L during intense exercise or prolonged hypoxia.^4,5^ This suggests that GPR81 is either physiologically quiescent basally or that it is primarily responsive to the local, rather than circulating, concentration of lactate. In adipocytes, activation of GPR81 by L-lactate prevents lipid breakdown and promotes storage of energy-rich metabolites in adipocytes.^2,6^ Synthetic GPR81 agonists^7–9^ are potential therapies for dyslipidemia and inhibit lipolysis in cultured adipocytes and in vivo.

GPR81 is also expressed in brain, kidney, liver, skeletal muscle,^2,6^ and immune cells,^10^ but the function in nonadipose tissue is poorly defined and the limited data is somewhat contradictory. In mouse macrophages and human monocytes, GPR81 activation suppresses Toll-like receptor pathways, preventing NLRP3 (NACHT, LRR and PYD domains-containing protein 3) inflammasome activation and cell death.^11^ This mechanism appears protective, with receptor activation reducing tissue injury in models of hepatitis, pancreatitis,^11^ and colitis.^12^ GPR81 inhibition/knockdown has also been shown to be protective, enhancing neuron survival in cerebral ischemia^13^ and slowing cancer growth,^14,15^ partly due to effects on the vasculature. For example, GPR81 knockdown in a breast cancer cell line suppressed VEGF (vascular endothelial growth factor) and amphiregulin, retarding angiogenesis.^14^ Similarly, in the sensorimotor cortex of GPR81 knockout mice (*Gpr81*^−/−^), the induction of VEGF-A and increased capillary density by either treadmill exercise or subcutaneous lactate injections was absent.^16^ Ex vivo lactate infusion in the rat retina decreases capillary lumen diameter,^17^ suggesting vasomotor effects of GPR81 activation. Indeed, a small molecule GPR81 agonist, AZ2 (aka AZ′5538), which suppressed in vivo free fatty acid levels, also increased blood pressure (BP) when infused intravenously.^9^ Nonselective α-adrenoceptor antagonism and endothelin receptor blockade separately reduced this pressor effect.^9^

The concept that lactate could influence blood pressure (BP) through activation of GPR81 is relevant to human health. In the current study, we used AZ′5538 and *Gpr81*^−/−^ mice to resolve the effects of GPR81 activation on BP and renal hemodynamics in mice and further demonstrated that genetic deletion of the receptor conferred protection against renal ischemia-reperfusion injury.

## Methods

See in the Data Supplement for detailed methods. The data that support the findings of this study are archived in the University of Edinburgh data storage and available from the corresponding author upon reasonable request.

Experiments were performed on adult male C57Bl/6JCrl (Charles River, Paris, France) or adult male *Gpr81*^+/+^ (wild-type) and *Gpr81*^−/−^ (knockout) mice on a C57BL/6JOlaHsd background.^9^ Experiments were performed under a UK Home Office Licence following ethical review by the University of Edinburgh.

### In Vivo Measurement of BP and Renal Function

In anesthetized mice, either AZ′5538 (1 µmol/kg bw/min) or 5% d-mannitol vehicle was infused intravenously for 15 minutes. BP was measured via a carotid cannula and renal hemodynamics measured via a Doppler transit time probe, and Doppler flux probes were inserted into the cortex and medulla. Separately, renal artery blood flow (RBF) was measured noninvasively by Pulse-wave Doppler (Vevo770 and 707B 30 MHz ultrasound probe; VisualSonics, Canada). Glomerular filtration rate (GFR) was measured by Fluorescein isothiocyanate-inulin clearance before and after administration of AZ′5538.

### In Vivo Blockade of Endothelin Receptors

Anesthetized C57Bl/6JCrl mice had separate intravenous infusion of AZ′5538 and endothelin receptor antagonists or their corresponding vehicles. Bosentan, a mixed endothelin receptor antagonist, was used at 20 and 40 mg/kg; BQ123 (endothelin-A receptor antagonist) and BQ788 (endothelin-B receptor antagonist) were used at 1 and 2 mg/kg.

### Ischemia-Reperfusion Injury (IRI)

Male *Gpr81*^−/−^ and wild-type littermates were subject to 27 minutes of renal pedicle clamping (reperfusion was confirmed visually) followed by nephrectomy of the nonclamped kidney. After 6 days, mice were killed by cervical dislocation and the kidney taken for mRNA extraction and analysis.

### RNA Analysis

Polymerase chain reaction (PCR) was used to determine if *Gpr81* was expressed in arteries. Subsequently, RNAscope in situ hybridization was used to localize *Gpr81* in artery and kidney sections. For quantitative PCR, RNA was extracted from quarter kidneys (RNeasy Mini Kit, Qiagen) and used to assess mRNA abundance by real-time quantitative PCR (Universal Probe Library; Sigma Aldrich). To assess *Gpr81* expression in defined populations of renal cells, kidneys from male mice were dissociated into a single-cell suspension and incubated with the following rat anti-mouse antibodies: PDGFRβ (Platelet Derived Growth Factor Receptor Beta), CD31 (cluster of differentiation 31), LTL (*Lotus tetragonolobus* lectin), and F4/80. Fluorescence-activated cell sorting was performed using the fluorescence-activated cell sorting Aria II (BD Biosciences) using 4′,6-diamidino-2-phenylindole to determine live cells. Cells were sorted into lysis buffer and RNA extracted using RNeasy microkit (Qiagen), quality checked by Agilent Bioanalyser (RNA integrity number >8), and amplified cDNA made from the RNA using Ovation RNA-Seq System V2 (NuGen).

### Endothelin-1

ET-1 (endothelin-1) was measured by ELISA (R&D Systems, United Kingdom). ET-1 protein concentrations were normalized to total protein (Pierce BCA assay; Thermo Fisher, United Kingdom).

### Statistics

All data are mean±SD. After confirming normal distribution, statistical comparisons (Graphpad Prism 6, La Jolla, CA) were made by using 1-sample *t* test (comparing against a value of 0), unpaired *t* test, and 1- or 2-way ANOVA. For 2-way ANOVA, the main effects of the genotype/treatment and time were assessed, and the interaction between them. Planned comparisons were made using Holm-Sidak with a family *P* value fixed at 0.05.

## Results

### Activation of GPR81 Increases BP and Decreases RBF

**Figure 1.**
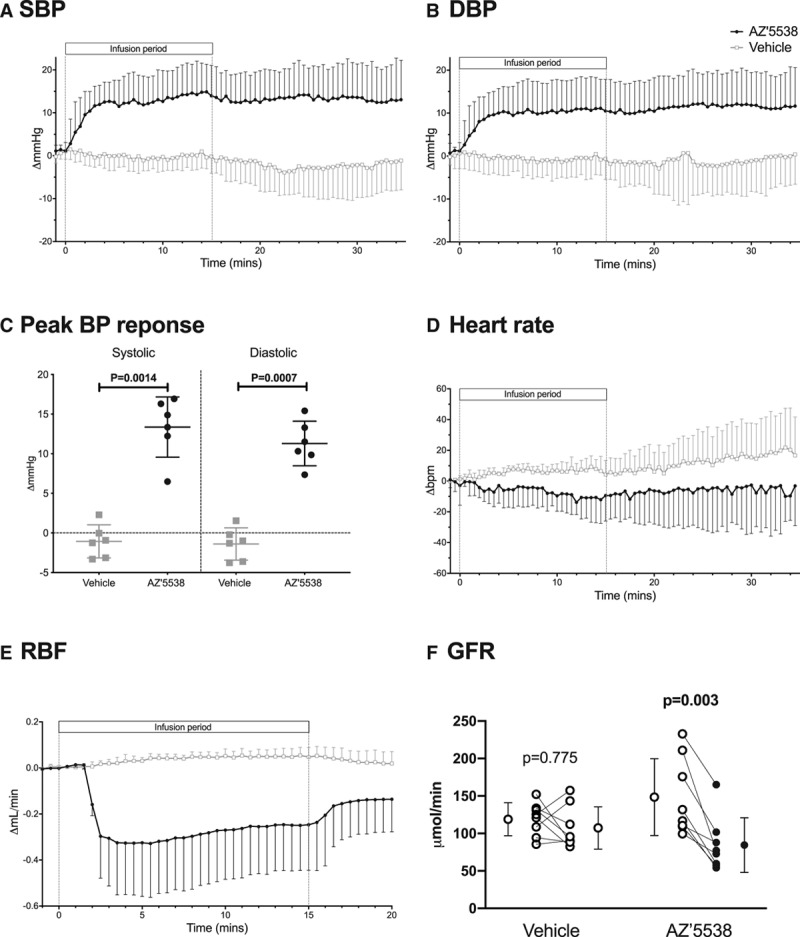
Effect of AZ′5538 infusion on blood pressure (BP) and renal hemodynamics. Mice were infused intravenously with vehicle (5% mannitol; n=6, open squares) or AZ′5538 (1 µmol/[kg·min]; n=6 black circles) for 15 minutes. Systolic blood pressure (**A**; SBP), diastolic blood pressure (**B**; DBP), peak BP responses (**C**), and heart rate (**D**). **E**, Renal artery blood flow (RBF) in separate mice infused with vehicle (n=6) or AZ′5538 (n=6). **F**, Glomerular filtration rate (GFR) before and after infusion with AZ′5538 (n=8), compared to vehicle (n=8). Data are mean±SD, and for **C** and **F**, individual datapoints are shown. Statistical comparisons were made by 2-way ANOVA for the main effects of treatment, of time and of the interaction (see text for *P* values) and by unpaired *t* test (**C** and **F**), with *P* values as shown.

In anesthetized male C57BL/6J mice, baseline systolic BP (SBP) was 86±9 mm Hg, diastolic BP (DBP) was 69±11 mm Hg, and heart rate was 284±38 bpm. AZ′5538 increased SBP (Figure 1A; ANOVA effect of treatment *P*=0.0013; effect of time and interaction *P*<0.0001) and DBP (Figure 1B; ANOVA effect of treatment *P*=0.0094; effect of time and interaction *P*<0.0001). The peak BP increases were 13±4 mm Hg and 11±3 mm Hg for SBP and DBP, respectively (Figure 1C). Heart rate fell with infusion of AZ′5538 compared with 5% mannitol vehicle (Figure 1D). All mice received a second infusion of AZ′5538, 35 minutes after the first administration. The pressor response to the second administration was attenuated, particularly in DBP (Figure S1 in the Data Supplement).

RBF was measured in 2 different groups of mice by direct Doppler ultrasound with a probe around the right renal artery (baseline RBF =0.60±0.23 mL/min) and by pulse-wave Doppler (baseline velocity =275±21 mm/s). AZ′5538 infusion significantly decreased RBF, by ≈50% measured by flow probe (Figure 1E; ANOVA effect of treatment *P*=0.027; effect of time and interaction *P*<0.0001) and by ≈30% by pulse-wave Doppler (Figure S2; peak decrease of −186±26 mm/s; ANOVA drug treatment *P*=0.0002, time and interaction *P*<0.0001). In another experiment, GFR was measured, and the mice were then randomized to receive continued intravenous vehicle (n=8) or AZ′5538 (n=8). There was no significant change in GFR with vehicle but AZ′5538 infusion caused GFR to fall significantly (Figure 1F).

### The Cardiovascular Effects of AZ′5538 Are Mediated Via *Gpr81*

**Figure 2.**
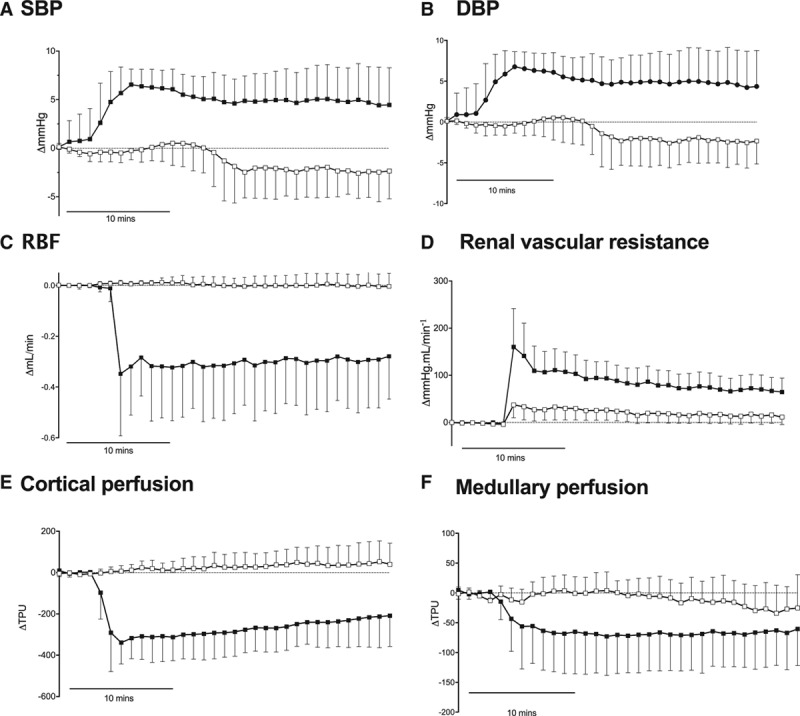
Blood pressure and hemodynamic effects of AZ′5538 are GPR81 (G-protein-coupled receptor 81) dependent. *Gpr81*^−/−^ mice (open circles; n=6) and wild-type littermates (closed squares; n=6) were infused intravenously with vehicle (5% mannitol) or AZ′5538 (1 µmol/[kg·min]; n=6 black circles) for 15 minutes. Systolic blood pressure (SBP, **A**), diastolic blood pressure (DBP, **B**), renal artery blood flow (RBF, **C**), renal vascular resistance (**D**), perfusion of the renal cortex (**E**), and perfusion of the renal medulla (**F**) ΔTPU= change in Total Perfusion Units. All data are mean±SD from baseline. Statistical comparisons were made by 2-way ANOVA for the main effects of genotype, time, and of the interaction (see text for *P* values).

BP and heart rate were comparable in *Gpr81*^−/−^ mice and wild-type littermates (Table S3). In wild types, AZ′5538 significantly increased SBP (Figure 2A; *P*<0.0001 for main effects of genotype and time and for interaction) and DBP (Figure 2B; *P*<0.0001 for main effects of genotype and time and for interaction) and decreased heart rate (Figure S3; ANOVA genotype *P*=0.025, time *P*=0.003, and interaction *P*<0.0001), consistent with our data in C57BL/6J mice. AZ′5538 did not increase BP in *Gpr81*^−/−^ mice.

RBF decreased significantly with AZ′5538 infusion in wild-type mice but remained unchanged in *Gpr81*^−/−^ mice (Figure 2C; ANOVA genotype *P*=0.008 interaction and time *P*<0.0001). Renal vascular resistance did not differ between genotypes, although the interaction was significantly different (Figure 2D; ANOVA genotype *P*=0.151, time *P*=0.022, and interaction *P*=0.0002). AZ′5538 reduced renal cortical perfusion but only in wild-type mice (Figure 2E; genotype *P*=0.0003 and time interaction *P*<0.0001). Medullary flux also fell in wild-type mice but not in the *Gpr81*^−/−^ (Figure 2F; genotype *P*=0.070, time and interaction *P*<0.0001). Baseline GFR was not different between genotype. AZ′5538 reduced GFR in wild-type mice (Figure 2G; ΔGFR=−125±48 µL/min; *P*=0.001 by 1-sample *t* test; n=6) but not in *Gpr81*^−/−^ mice (Figure 4; ΔGFR=−39±83 µL/min; *P*=0.303; n=6).

### Expression of *Gpr81* in Arteries and Kidney

**Figure 3.**
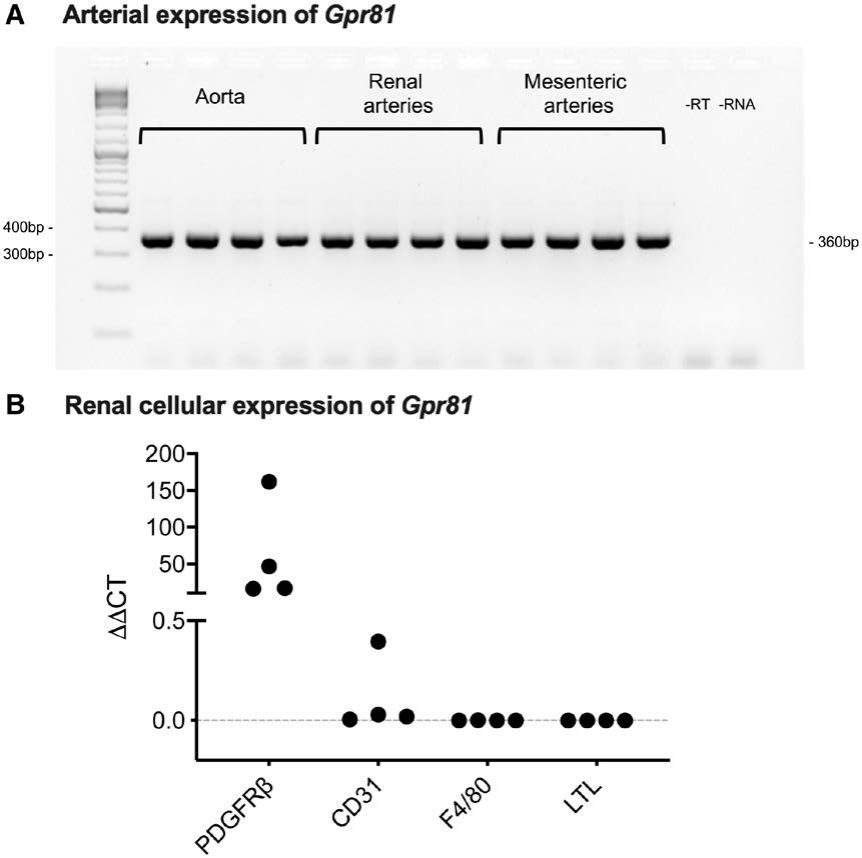
*Gpr81* (G-protein-coupled receptor 81) expression in artery and renal cells. **A**, RNA was extracted and reverse transcribed from C57Bl/6JCrl mouse vessels. Negative controls were samples where reverse transcription enzyme, or RNA, were left out at the cDNA conversion step. All samples underwent end point polymerase chain reaction (PCR) before gel electrophoresis. n=4 for all vessel types, pooled from 2 mice for renal arteries and mesenteric arteries. **B**, Defined populations of cells were isolated by fluorescence-activated cell sorting (FACS) from kidney taken from n=4 mice. Quantitative PCR was used to measure *Gpr81* in PDGFRβ^+^ (pericytes), CD31^+^ (endothelial cells), F4/80^+^ (macrophages), and LTL^+^ (tubule) cell groups. Expression was significantly >0 in PDGFRβ^+^ cells (*P*<0.001).

**Figure 4.**
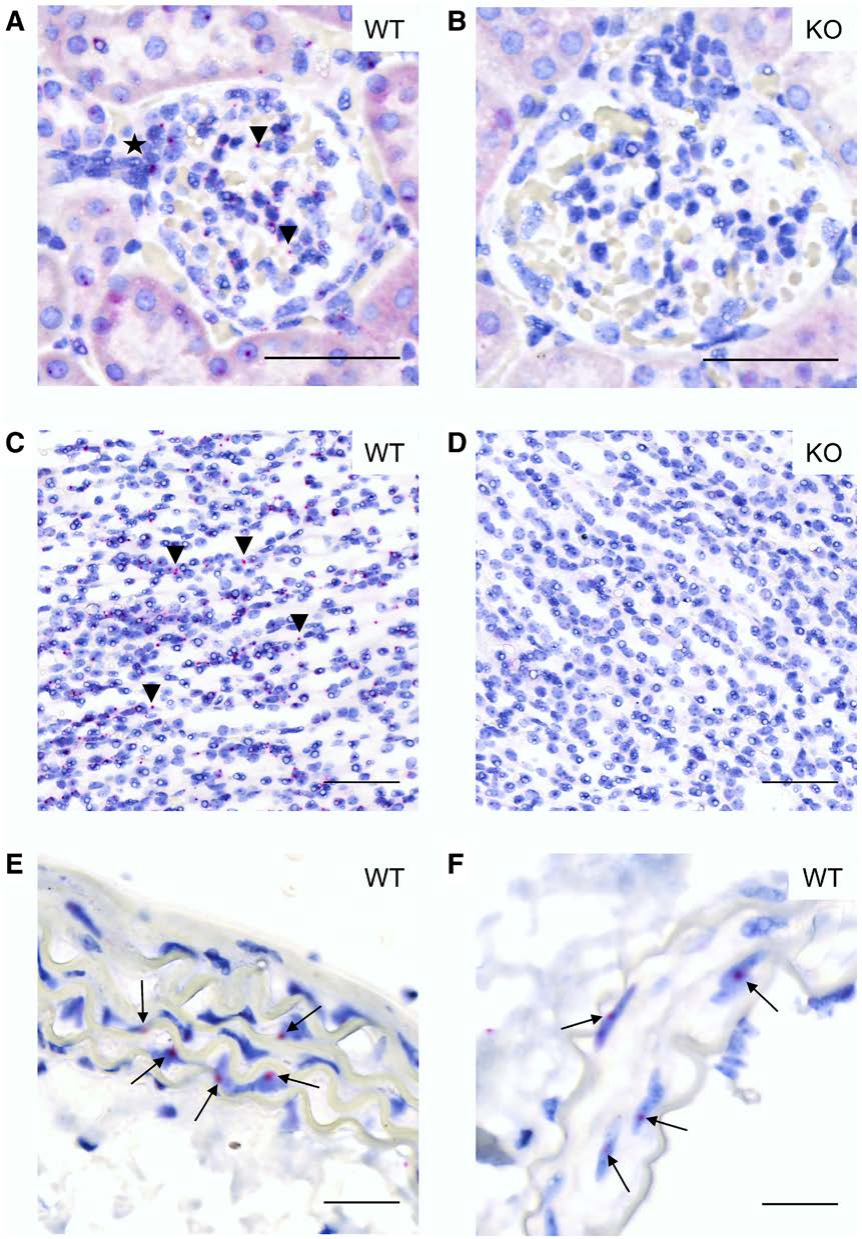
Representative figures of *Gpr81* in situ hybridization. Positive *Gpr81* mRNA expression shown by red punctuated dots. Expression found in wild-type (WT) mouse glomeruli of the kidney cortex (**A**) where the star indicates an arteriole and medulla (**C**). No staining was seen in *Gpr81*^−/−^ mouse kidney tissues (**B** and **D**). Receptor expression also seen in smooth muscle cells of the WT mouse aorta (**E**) and renal artery (**F**) where staining is indicated with arrows. Scale bars are 50 μm (**A–D**) and 20 μm (**E** and **F**).

*Gpr81* was expressed in aorta, renal, and mesenteric arteries (Figure 3A). Separately, quantitative PCR was used to assess *Gpr81* expression in fluorescence-activated cell sorting–isolated renal cell populations (Figure 3B; n=4 mice). *Gpr81* mRNA was highly expressed in PDGFRβ^+^ cells (pericytes) from all 4 samples, identified in only one of 4 samples of CD31^+^ cells (endothelial) and was undetectable in LTL (renal tubules) and F4/80 (macrophage) cell populations. Using in situ hybridization on whole kidney sections, *Gpr81* was localized in the cortex and medulla of wild-type but not *Gpr81*^−/−^ mice (Figures 4A through 4D). The staining in the cortex was localized mainly to the glomeruli (Figure 4A and Figure S4), particularly at the vascular pole, consistent with localization in arterioles. *Gpr81* was also expressed in the medulla (Figure 4C) and did not co-localize with the nuclear stain. We examined *Gpr81* expression in aorta and renal artery (Figure 4E and 4F), observing positive staining in the medial layer, indicating localization to vascular smooth muscle cells. No staining was visible in arteries from *Gpr81*^−/−^ mice (Figure S5).

### Cardiorenal Effects of GPR81 Activation Are Endothelin-1 Dependent

**Figure 5.**
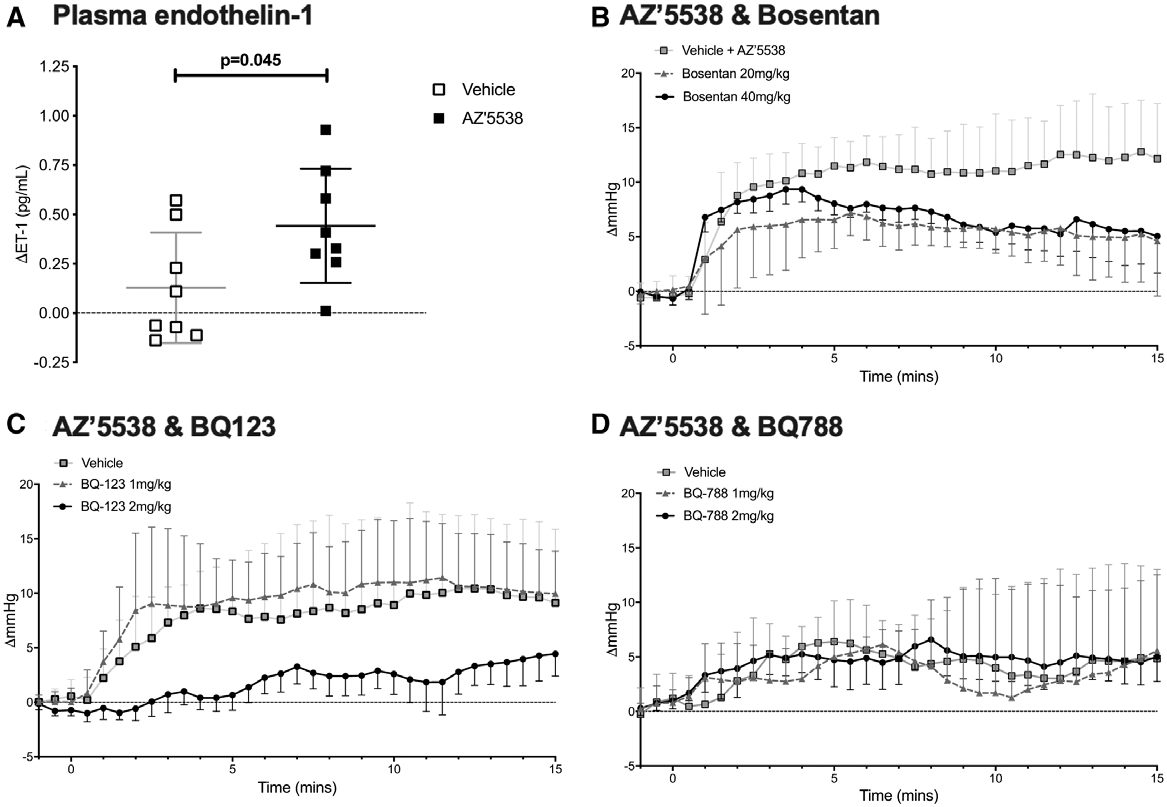
Role of the endothelin system. **A**, The change in plasma endothelin-1 in C57Bl/6J mice infused with either vehicle (open square; n=8) or AZ′5538 (1 µmol/[kg·min] black square; n=8) for 15 min. Individual data points and mean±SD are shown. In separate experiments, C57Bl/6J was infused intravenously with vehicle or an endothelin receptor antagonist for 25 min before treatment with AZ′5538 (1 µmol/[kg·min] for 15 min). The change in systolic blood pressure over baseline is shown. **B**, Bosentan; **C**, BQ123; and **D**, BQ788. Data are mean±SD, analyzed by 2-way ANOVA. Statistical comparisons were made by 2-way ANOVA for the main effects of treatment, time, and of the interaction (see text for *P* values).

AZ′5538 infusion significantly increased plasma ET-1 concentration (Figure 5A) without changing the amount in aorta or whole kidney homogenates (Figure S6); renal expression of *Edn1* was lower in mice that had received a 15-minute infusion of AZ′5538 (Figure S6). To assess functional crosstalk between GPR81 and the endothelin system, wild-type mice were pretreated with bosentan, before infusion of AZ′5538. Bosentan pretreatment at 40 mg/kg did not change the peak pressor response to AZ′5538 but significantly blunted the sustained effect on BP (Figure 5B; treatment *P*=0.024, time *P*<0.0001, and interaction *P*=0.064); similar actions were also observed at the 20 mg/kg bosentan. Next, we used pretreatment with BQ123 and, separately, BQ788. Baseline BP was not changed by either BQ123 or BQ788 (Figure S7). BQ123 at 2 mg/kg largely prevented the pressor effect of GPR81 activation, and there was no sustained BP rise in this group (Figure 5C and Figure S8; BQ123 treatment *P*=0.044, time *P*<0.0001, and interaction *P*=0.988). BQ788 did not change the BP response to AZ′5538 (Figure 5D and Figure S8).

### *Gpr81*^−/−^ Mice Are Protected From Renal Ischemia-Reperfusion Injury

**Figure 6.**
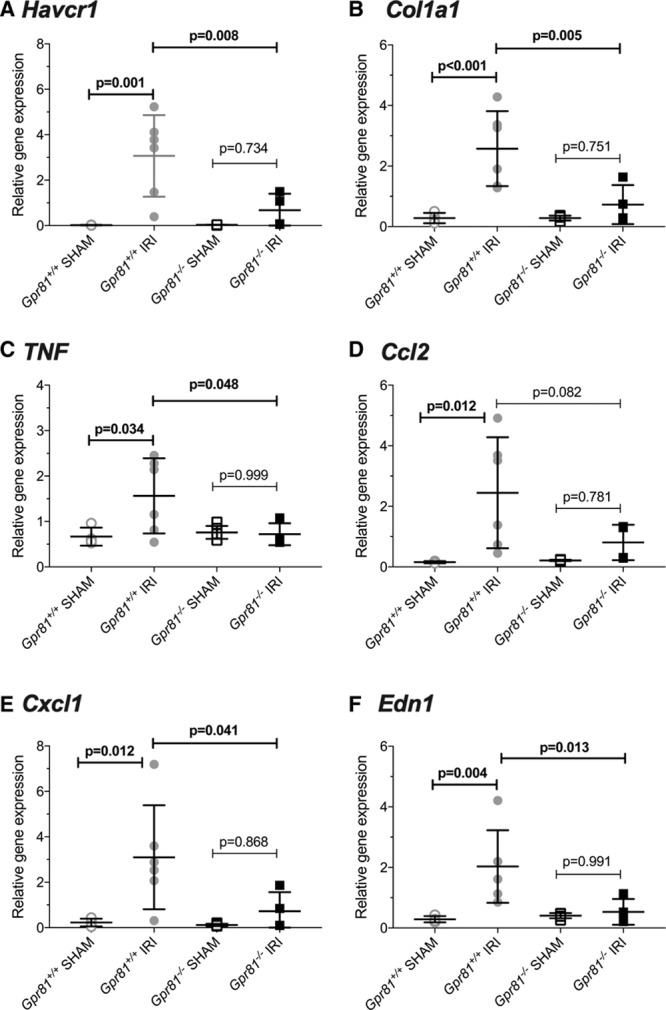
*Gpr81*^−/−^ mice have reduced injury following renal ischemia-reperfusion. Renal ischemia-reperfusion injury or a sham operation was performed on *Gpr81*^−/−^ (n=4/6) and wild-type mice (n=6/4). One week later, the renal expression of the following genes was measured by quantitative polymerase chain reaction: (**A**) *Havcr1* (encoding KIM-1 [kidney injury molecule 1]); (**B**) *Col1a1* (encoding collagen type 1 α1); (**C**) *Tnf* (encoding TNF-α [tumor necrosis factor-α], ); (**D**) *Ccl2* (encoding MCP-1 [monocyte chemotactic protein 1]); (**E**) *Cxcl1* (encoding C-X-C motif chemokine ligand 1); and (**F**) *Edn1* (encoding preproendothelin-1). Expression is normalized to housekeepers; individual data points and group mean±SD are shown. Statistical comparisons were made by 1-way ANOVA with Holm-Sidak test for planned comparisons with *P* values as indicated.

In wild-type mice, renal ischemia significantly increased the mRNA expression of the tubule injury marker KIM-1 (Kidney Injury Molecule 1) (Figure 6A), the fibrosis marker collagen 1a1 (Figure 6B), the inflammatory cytokines TNF-α (Tumor necrosis factor alpha; Figure 6C), MCP-1 (monocyte chemotactic protein 1; Figure 6D), CXCL-1 (C-X-C Motif Chemokine Ligand-1; Figure 6E), CXCL-10 (C-X-C Motif Chemokine Ligand-10; Figure S9A), and the pan-macrophage marker F4/80 (Figure S9B). ET-1, assessed by renal expression of *Edn1* mRNA, was also increased by injury (Figure 6F). The transcriptional response to IRI was significantly blunted in *Gpr81*^−/−^ mice. Injury did not affect endothelin-A receptor expression in either genotype (Figure S9C); endothelin-B receptor expression was reduced in both genotypes by IRI (Figure S9D).

## Discussion

A decade ago, the orphan receptor GPR81 was shown to be activated with low affinity by L-lactate and α- and γ-hydroxybutyrate.^6^ *GPR81* mRNA was enriched in mouse and human adipocytes and receptor activation by lactate inhibited lipolysis.^6^ *GPR81* mRNA expression was also found in nonadipose tissue, with expression in the highly vascularized tissues of heart, skeletal muscle, and kidney being ≈10% of that in adipocytes.^6^ Here, we detected *GPR81* mRNA in whole kidney homogenates. In each of 3 artery types examined, *GPR81* localized to the smooth muscle layer. Transgenic fluorescent-reporter mice show *GPR81* expression in the vessel wall of pial arteries.^16^ Single-cell RNA sequencing detected *GPR81* in cerebral and lung vascular smooth muscle cells.^18^ *GPR81* has been identified in cultured human umbilical vein endothelial cells,^19^ but we suggest this is not a major expression site, finding discernable levels in only 1 of 4 isolated renal endothelial cell samples and no in situ endothelial *GPR91* mRNA expression in large arteries. Similarly, *GPR81* does not co-localize with endothelial cell markers in the cerebral vasculature.^16^ Within the kidney, *GPR81* was additionally expressed in glomerular arterioles, with a previous study reporting afferent arteriolar localization in dog and mouse.^9^ We detected *GPR81* in perivascular cells, particularly, in the renal medulla and in isolated PDGFRβ^+^ cells. *GPR81* also colocalizes with PDGFRβ-expressing cells and leptomeningeal cells in the cerebral microcirculation.^16^

This expression profile suggests that GPR81 is intimately involved in vascular/microvascular function, as described for other metabolic GPCRs. For example, activation of GPR109a by niacin reduces reactive oxygen production in arterial endothelial cells^20^ and promotes vasodilation by stimulating prostaglandin production.^21^ Succinate, which activates GPR91, acutely increases circulating Ang II (angiotensin II) and BP in rats.^22^ Like GPR81, GPR91 is also coupled to G_i_, and succinate has direct effects on arterial contractility.^23^ For lactate/GPR81, however, functional data are limited. Increasing lactate concentration in the brain, either through exercise or by exogenous administration, promotes angiogenesis.^16^ This effect is GPR81-dependent since lactate-induced angiogenesis does not occur in *Gpr81*^−/−^ mice. Lactate also constricts retinal microvessels, but it is not known whether this is GPR81–mediated.^17^ Infusion of very high concentrations of lactate increases BP in rats,^24^ most likely reflecting panic-induced sympathoexcitation, rather than activation of GPR81.

The recent development of potent GPR81 agonists to treat dyslipidemia^8,9,25^ provides tools to probe the cardiovascular physiology of GPR81. One study has examined this, finding that structurally distinct GPR81 agonists increased SBP by ≈15 mm Hg in rats and dogs when given intravenously and by ≈5 mm Hg in mice when given orally.^9^ Our studies confirm and extend this work, unequivocally demonstrating that one of these compounds, AZ′5538, increases BP dependent on the expression of GPR81. We further show that GPR81 activation reduces renal artery flow, cortical and medullary perfusion, and GFR. The rapid increase in BP makes it difficult to unambiguously interpret these renal hemodynamic effects of AZ′5538. However, RBF and cortical perfusion normally autoregulate when BP increases,^26,27^ and our data most likely reflect direct vasoconstriction of the renal artery and preferential constriction of the afferent over efferent arteriole, accounting for the GFR reduction. Furthermore, expression of GPR81 in renal PDGFRβ cells is consistent with localization in pericytes, contractile cells which regulate vasa recta blood flow,^28^ independent of changes in total or cortical blood flow.^29^ Thus, pericyte constriction may contribute to reduced intrarenal perfusion following GPR81 activation, but this was not demonstrated directly.

The cellular mechanism underpinning GPR81-mediated vasoconstriction is not fully known. In adipocytes, GPR81 couples to G_i_, downregulating cAMP production and protein kinase A signaling.^30^ Activation of G_i_ pathways in vascular smooth muscle cells, by GPCR kinase 5, for example, lowers intracellular cAMP (Cyclic adenosine monophosphate), enhances vasoconstriction, and causes sustained hypertension.^31^ Similarly, activation of A1 receptors by adenosine constricts the renal afferent arteriole by a G_i_-mediated cascade involving activation of phospholipase C.^32^ Reciprocally, agents that increase cAMP promote arterial vasorelaxation.^33,34^ However, our data do not support a direct vasomotor effect of GPR81 activation and instead indicate dependency on ET-1 release and subsequent endothelin-A receptor activation. Notably, ET-1 is synthesized by arterial myocytes,^35^ a cell that expresses GPR81. Further, a reduction in cAMP stimulates the production of ET-1 by myocytes,^36–38^ as does vascular disease and injury.^39,40^ The hemodynamic response to AZ′5538 is also consistent with this sequence: ET-1, via endothelin-A receptors, induces a stronger constriction of the afferent than efferent arteriole^41^ and also causes pericyte contraction, reducing vasa recta blood flow,^42^ both of which were found in our study.

In the final series of experiments, renal IRI was induced as this is known to increase intrarenal extracellular lactate.^43^ We selected a panel of transcriptional markers to capture cardinal features of IRI, tubular injury (KIM-1), increased matrix deposition (collagen 1a1), enhanced intrarenal cytokine production (TNF-α, CXCL-1, CXCL-10), and increased monocyte/macrophage infiltration (F4/80). In wild-type mice, ischemia-reperfusion increased expression of these markers, as anticipated. In contrast, this transcriptional response to ischemia-reperfusion was absent or blunted in *Gpr81*^−/−^ mice. Similar outcomes are reported in cerebral ischemic injury: 3-hydroxy-butyrate, which antagonizes GPR81, prevents lactate-induced injury in primary cultured neurons and is neuroprotective in mice exposed to cerebral artery occlusion.^13^ In contrast, overexpression of GPR81 amplifies sensitivity to hypoxic injury in a neuronal cell line.^13^

We cannot unequivocally establish the mechanism of renoprotection in *Gpr81*^−/−^ mice, but these animals did not display the sustained post-ischemic increase in renal ET-1 expression that occurred in wild-type mice. This is likely to be important for at least 2 reasons. First, endothelin-A receptor–dependent vasoconstriction may contribute to progressive renal injury, which has a strong hemodynamic component.^44^ Second, ET-1, via endothelin-B receptors, can drive epithelial-mesenchymal transition and promote renal fibrosis.^45^ *Gpr81*^−/−^ mice did not respond to ischemia-reperfusion with an increase in TNF-α or collagen 1a1 production, and the disconnection between extracellular lactate and ET-1 may account for this.

A limitation of our work is that all of the studies were performed on male mice. Although recent studies show that sex does not influence afferent arteriolar reactivity to ET-1,^46^ other literature indicates sex differences in the renal actions of ET-1, which might be particularly relevant in an injury context.^47^ For example, post ischemia-reperfusion, male rats display an exaggerated early increase in renal vascular resistance and then a more pronounced decline in renal function and lower survival rate than do female rats.^48^ Furthermore, IRI increased ET-1 expression in male rats and prophylactic endothelin-A blockade improved survival.^48^ In marked contrast, endothelin-A receptor blockade in females was detrimental, worsening post-ischemic survival rates, suggesting a protective role of ET-1/endothelin-A receptors activation. Overall, these data indicate that female *Gpr81*^−/−^ may not show the post-ischemic renoprotection of male animals identified by the current study.

## Perspectives

In summary, our study indicates that GPR81 activation regulates macro- and microvascular perfusion within the kidney, dependent on ET-1 signaling. The physiological requirement for a system that would lead to vasoconstriction of regions with high anaerobic cellular metabolism is not readily apparent, given the injurious effect of hypoxia. However, earlier work in skeletal muscle associated exercise-induced accumulation of extracellular lactate with the release of ET-1 and proposed that constraining vasodilation of the skeletal vasculature would help maintain systemic BP during exercise.^49^ It may be that GPR81 activation similarly constrains the propensity to increase flow into relatively ischemic areas of the kidney and thereby militates against hyperemic damage. Additionally, the ET-1 release may contribute to vascular remodeling with sustained hypoxia. Overall, our data suggest that blockade of the GPR81/ET-1 system could offer beneficial vascular support in the post-injury phase.^50^

## Acknowledgments

We thank the University of Edinburgh The Queen’s Medical Research Institute Flow Cytometry and Cell Sorting Facility and acknowledge Kristina Wallenius and Robert Unwin (AstraZeneca R&D Gothenburg) for data discussions and for providing us with samples of kidney tissue from the IRI experiment.

## Sources of Funding

This work was funded by a PhD studentship from the British Heart Foundation (BHF) (FS/15/63/32033) and an Intermediate Fellowship from Kidney Research United Kingdom (PDF6/2012). Abstracts of this work have been presented at Experimental Biology 2017, Europhysiology 2018 and Experimental Biology 2019, with travel support to N.K. Jones from the Physiological Society and the BHF Centre of Research Excellence Edinburgh (RE/13/3/30183).

## Disclosures

M.A. Bailey discloses prior research funding from AstraZeneca (Research Agreement 10028531). AZ′5538 compound and *GPR81* knockout mouse for this study were provided free of charge by AstraZeneca. R.I. Menzies is now employed by AstraZeneca (BioPharmaceuticals R&D, AstraZeneca, Gothenburgh, Sweden). The other authors report no conflicts.

## Supplementary Material



## Novelty and Significance

### What Is New?

- Activation of GPR81 (G-protein-coupled receptor 81) increases blood pressure, induces renal vascular and microvascular constriction, and reduces glomerular filtration rate.
- The systemic and renal hemodynamic effects are mediated by release of ET-1 (endothelin-1) and activation of the endothelin-A receptor.
- *Gpr81* is localized to arterial vascular smooth muscle, to the arterioles, and in pericytes that influence capillary blood flow.
- Genetic deletion of *Gpr81* in mice protects against renal ischemic injury.

### What Is Relevant?

- The endogenous ligand for GPR81 is lactate. Our results suggest that lactate, via activation of GPR81, may contribute to the physiological regulation of blood pressure and renal hemodynamics.
- GPR81 antagonists may offer a route for vascular support in renal ischemia, the most common cause of acute kidney injury in humans.

### Summary

In male mice, activation of GPR81 increased blood pressure and reduced renal artery blood flow, renal capillary perfusion, and glomerular filtration rate. GPR81 activation rapidly increased plasma ET-1: the pressor effects were prevented by selective endothelin-A receptor antagonism. In male wild-type mice, ischemia-reperfusion increased renal expression of ET-1 and genes associated with injury, fibrosis, and inflammation. *Gpr81*^−/−^ did not show this post-ischemic injury response
