# Supplementary figures and images for: Endothelin-1 Mediates the Systemic and Renal Hemodynamic Effects of GPR81 Activation
Source: Hypertension. 2020 Mar 23;75(5):1213–22. doi: 10.1161/HYPERTENSIONAHA.119.14308 (PMC7176350; doi:10.1161/HYPERTENSIONAHA.119.14308)

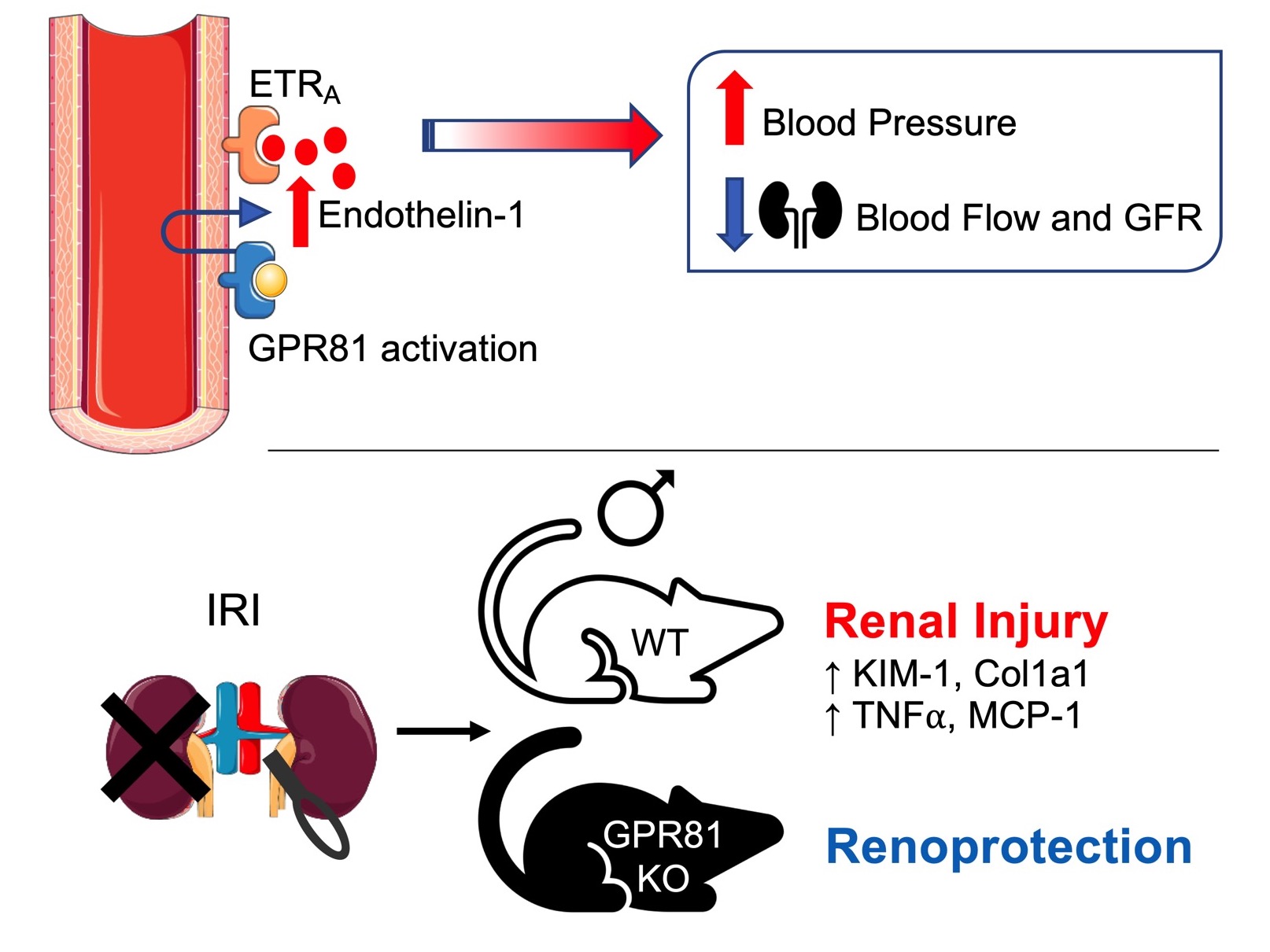

Supplement: Supplementary file 1 [file hyp-75-1213-s001.jpg]
