# Supplementary material for: Endothelin-1 Mediates the Systemic and Renal Hemodynamic Effects of GPR81 Activation
Source: Hypertension. 2020 Mar 23;75(5):1213–22. doi: 10.1161/HYPERTENSIONAHA.119.14308 (PMC7176350; doi:10.1161/HYPERTENSIONAHA.119.14308)

**ONLINE DATA SUPPLEMENT**

**Endothelin-1 mediates the systemic and renal hemodynamic effects of GPR81 activation**

Natalie K Jones^1^, Kevin Stewart^1^, Alicja Czopek^1^, Robert I Menzies^1^, Adrian Thomson^1^, Carmel M Moran^1^, Carolynn Cairns^1^, Bryan R Conway^1^, Laura Denby^1^, Dawn E.W. Livingstone^1^, John Wiseman^2^, Patrick W Hadoke^1^, David J Webb^1^, Neeraj Dhaun^1^, James W Dear^1^, John J Mullins^1^, Matthew A. Bailey^1^

^1^University/British Heart Foundation Centre for Cardiovascular Science, The University of Edinburgh, Scotland, UK, ^2^Discovery Sciences, IMED Biotech Unit, AstraZeneca R&D Gothenburg, Sweden

**Supplemental Methods**

Experiments were performed on adult male C57Bl/6JCrl (Charles River, Paris, France) or adult male *Gpr81*^+/+^ (wildtype) and *Gpr81^-/-^* (knockout) mice on a C57BL/6JOlaHsd background, generated by heterozygote crosses. Knockout and wildtype mice were genotyped by PCR and littermates were used. All mice were group housed at 22°C±1°C, 55% humidity, with a 12-hour light dark cycle (lights on at 07:00), in home cages provided with sawdust and standard bedding material and free access to standard rodent chow and water. Experiments were performed under a UK Home Office Licence according to the Animals (Scientific Procedures) Act and following ethical review by the University of Edinburgh. Following pilot experiments to parameterise the anticipated reponse, formal power calculations were performed against primary outcomes. Experiments reported here were performed under a single blind (to genotype/treatment), unless otherwise stated. For experiments on commercially sourced C57Bl/6JCrl, mice were randomised to treatment. Randomisation was not used for in vivo experiments on Gpr81^-/-^ mice.

**In vivo measurement of BP and renal function:** mice were anesthetized with sodium thiobutabarbital (Inactin, 120mg/kg IP; Sigma Aldrich, UK) between 10am and 2pm local time. The right jugular vein and carotid artery were cannulated for IV infusion and blood sampling/BP recording (Powerlab; AD Instruments, UK), respectively. A tracheotomy maintained a clear airway and core temperature was maintained at 37^o^C. After a 30-minute post-surgery stabilization period, either AZ’5538 (1µmol/kg bw/min; pH 5) or 5% D-mannitol vehicle (pH 5) was infused IV for 15 minutes. Mice therefore received ~450nmoles of AZ’5538, which was previously shown to have a EC_50_ of 180nmol/l against the mouse receptor and a blood t^1^/_2_ of 5 hours.^9^ BP was acquired at 1kHz; SBP and DBP were resolved post-acquisition using cyclic measurement function on LabChart 7 (ADInstruments, UK). For measurement of renal hemodynamics, a midline laparotomy was also performed and a Doppler transit time probe (Transonic, Ithaca, NY, USA) placed around the right renal artery using Surgilube gel (HR Pharmaceuticals, Pennsylvania, USA) for acoustic coupling. In some experiments, Doppler flux probes (Transonic) were inserted into the cortex and medulla of the right kidney and tissue perfusion measured during administration of AZ’5538 or vehicle as before. Probe position was confirmed post-mortem. In separate experiments, renal artery blood flow was measured non-invasively by ultrasound. Mice were anesthetized, the jugular vein cannulated as before and placed supine on a heat pad. Pulse-wave Doppler using a Vevo770 with a 707B 30MHz ultrasound probe (VisualSonics, Toronto, Canada) measured blood velocity from the right renal artery. Peak velocity, velocity time integral (VTI) and Pourcelot resistive index were all measured at baseline and over 5-minute periods during IV administration of AZ’5538 or vehicle as above. For glomerular filtration rate (GFR) measurements, mice were continuously infused (0.2mL/10g/hr) with a solution containing: 100mM NaCl, 5mM KCl, 15mM NaHCO_3_, and 0.25% FITC-inulin, pH 7.4). Urine and plasma were collected over a baseline period (5% D-mannitol infusion) and after administration of AZ’5538 (AZ’5538 at 1µmol/kg/min).

***In vivo blockade of endothelin receptors****:* C57Bl/6JCrl mice were anesthetized and surgically prepared with two IV lines permitting separate infusion of AZ’5538 and the endothelin receptor antagonists. Bosentan (Stratech Scientific, UK), BQ123 (Bio-Techne, UK); and BQ788, (Abcam, UK), or their corresponding vehicles (0.9% saline for bosentan and BQ123; 2.3% or 4.6% DMSO for BQ788) were infused IV at 0.2mL/10g/hr for 40 minutes, for the final 15 minutes, AZ’5538 was also infused as previously. Bosentan, a mixed endothelin receptor antagonist, was used at 20 and 40mg/kg; BQ123 (endothelin-A receptor antagonist) and BQ788 (endothelin-B receptor antagonist at 1 and 2mg/kg.

**Ischemia-reperfusion injury:** male *Gpr81^-/-^* and wild-type mice, aged 13-15 weeks, were anesthetized with isoflurane. A laparotomy was performed, and the left kidney was clamped at the renal pedicle for 27 minutes. The clamp was release and reperfusion was confirmed visually. The right kidney was removed and the wound closed. Animals were given analgesia during the post-operative recovery period. Two *Gpr81^-/-^* mice showed signs of post-surgical complications and were by killed by cervical dislocation <48h after the operation. These animals were not included in the analysis. The remaining animals were humanely killed at post-surgery day 5 and day 6 and the kidneys taken. These experiments were performed at AstraZeneca (Gothenburg, Sweden) and a pole of the kidney was sent to Edinburgh for mRNA extraction and analysis.

**PCR:** to determine if *Gpr81* was expressed in arteries***,*** frozen aorta (single), renal and mesenteric arteries (pooled from two mice) were crushed with mortar and pestle and RNA was isolated (RNeasy Micro Kit; Qiagen, Germany) and 300ng reverse transcribed (High Capacity cDNA Reverse Transcription kit, Applied Biosystems, USA) and then diluted 1:5 before PCR (VWR Red Taq DNA Polymerase Master Mix; Radnor, USA). Primer details are in Table S1. 10µL of each PCR product was electrophoresed alongside a 100bp DNA ladder (New England Biolabs, USA) on a 2% agarose gel for 1 hour 30 minutes at 80V.

For quantitative PCR, RNA was extracted from quarter kidneys (RNeasy Mini Kit, Qiagen) and 1µg reverse transcribed (High Capacity cDNA Reverse Transcription kit, Applied Biosystems) and diluted 1:40 before real time qPCR using the Universal Probe Library system (Sigma Aldrich). Primers and probe number are in Table S2. Three housekeeper genes were employed, *Rn18S*, *Hrpt* and *Tbp.* The expression of housekeepers was not different between genotype or treatment group and mean expression was used to normalize target gene expression.

**In situ hybridization**: Kidneys, aorta and renal arteries were dissected from *Gpr81^-/-^* and wild-type mice culled via asphyxiation with CO_2_. Samples were formalin-fixed in 10% neutral buffered formalin for 24 hours at room temperature before paraffin embedding and 5µm sections cut. Slides were baked for 2 hours at 60^o^C before undergoing the RNAscope 2.5 HD Red assay (Advanced Cell Diagnostics, UK) using a red labelled *Gpr81* probe alongside positive and negative control probes. Stained slides were then imaged by AxioScan (Zeiss, UK) with a 20x objective for kidney sections and 40x for vessels.

**RNA extraction from single populations of kidney cells:** Male C57BL6J mice (n=4) were euthanised via rising CO_2_ and perfused with 5mL ice-cold PBS through the heart. Kidneys were harvested, weighed, finely minced and placed in gentleMACS™ C Tubes with digestion buffer (Collagenase Type II 0.425mg/mL, Collagenase D 0.625mg/mL, Dispase 1mg/mL and DNAse 30μg/mL) and dissociated using the gentleMACS*™* Dissociator. Cellular suspensions were digested at 37⁰C for 30 mins then gentleMACS*™* dissociated for a second time. The cellular suspensions were put through 100μM, 70μM and 40μM sieves sequentially and red blood lysis performed with Red Blood Cell Lysing Buffer (Sigma). The single-cell suspension was incubated with the following rat anti-mouse antibodies: PDGFRβ (clone APB5, Biolegend 1:100), CD31, (clone 390, Biolegend, 1:200), LTL (*Lotus tetragonolobus* lectin, VectorLabs, 1:400) and F4/80 (Clone BM8, dilution 1:100, ThermoFisher)*.* Fluorescence activated cell sorting (FACS) was carried out using the FACS Aria II (BD Biosciences) using DAPI to determine live cells. Cells were sorted into RLT-plus buffer and RNA extracted using RNeasy microkit (Qiagen), quality checked by Agilent Bioanalyser (RIN>8) and amplified cDNA made from the RNA using Ovation RNA-Seq System V2 (NuGen).

**Endothelin-1 measurement:** Plasma samples were taken from C57BL6J mice at baseline and after 15-minute infusion of either vehicle or AZ’5538, as above. Mice were culled and tissue taken post-mortem. The expression of ET-1 in plasma, kidney and aorta was measured by ELISA (R&D Systems, UK). ET-1 protein concentrations were normalized to total protein (Pierce BCA assay; Thermo Fisher, UK).

**Table S1. End point PCR primers.** Primers used to determine Gpr81 expression in aorta, mesenteric and renal arteries of wild type C57 mice.

| Gene name | Protein | Forward Primer | Reverse Primer | Product size |
| --- | --- | --- | --- | --- |
| *Hcar1/Gpr81* | GPR81 | ttggagatatcgcctgtcgc | ggctccaaacaacgttgacc | 360bp |

**Table S2 – Real time Universal Probe Library PCR primers**

| Coded protein | Gene Name | Forward Primer | Reverse Primer | UPL probe number |
| --- | --- | --- | --- | --- |
| 18S | *Rn18s* | gccgctagaggtgaaattctt | cgtcttcgaacctccgact | 93 |
| COL1A1 | *Col1a1* | ccgctggtcaagatggtc | ctccagcctttccaggttct | 1 |
| CXCL1 | *Cxcl1* | agactccagccacactccaa | tgacagcgcagctcattg | 83 |
| CXCL10 | *Cxcl10* | aatgaaagcgtttagccaaaaa | aggggagtgatggagagagg | 56 |
| ET-1 | *Edn1* | tccttgatggacaaggagtgt | cccagtccatacggtacga | 29 |
| ETRA | *Ednra* | tgtgagcaagaaattcaaaaattg | atgaggcttttggactggtg | 34 |
| ETRB | *Ednrb* | tcagaaaacagccttcatgc | gcggcaagcagaagtagaaa | 83 |
| F4/80 | *Adgre1* | aggaggacttctccaagccta | aggcctctcagacttctgctt | 42 |
| GPR81 | *Hcar1* | ggtggcacgatgtcatgtt | gaccgagcagaacaagatgatt | 4 |
| HPRT | *Hprt* | tcctcctcagaccgctttt | aacctggttcatcatcgctaa | 95 |
| KIM1 | *Havcr1* | tccacacatgtaccaacatcaa | gtcacagtgccattccagtc | 25 |
| MCP-1 | *Ccl2* | catccacgtgttggctca | gatcatcttgctggtgaatgagt | 62 |
| TBP | *Tbp* | gggagaatcatggaccagaa | gatgggaattccaggagtca | 97 |
| TNFα | *Tnf* | agcctcttctcattcctgctt | atgagagggaggccatttg | 49 |

**Table S3. Cardiovascular and renal parameters in** **Gpr81^+/+^ and Gpr81^-/-^ mice.** Systolic blood pressure (SBP), diastolic blood pressure (DBP), heart rate (HR), renal blood flow (RBF; left kidney) and glomerular filtration rate (GFR; both kidneys) in Gpr81^+/+^ and Gpr81^-/-^ littermates under anaesthesia. Data are mean±SD analysed by unpaired t-test. For SBP, DBP and HR n=10 per genotype. RBF and GFR were measured in separate groups of mice (n=5 for RBF; n=6 for GFR per genotype).

|  | *Gpr81^+/+^* | *Gpr81^-/-^* | P |
| --- | --- | --- | --- |
| SBP (mmHg) | 73±19 | 71±17 | 0.7479 |
| DBP (mmHg) | 60±14 | 60±17 | 0.9895 |
| HR (bpm) | 281±58 | 293±48 | 0.6119 |
| RBF (mL/min) | 0.63±0.40 | 0.48±0.18 | 0.4609 |
| GFR (μL/min) | 270±70 | 289±44 | 0.5874 |

**Figure S1. A second infusion of AZ'5538 has a blunted pressure response.** All data are mean±SD, n=6. C57BL/6J mice were infused with AZ’5538 for 15 minutes with a 20-minute window of no infusion. Traces show A) systolic and B) diastolic blood pressure.


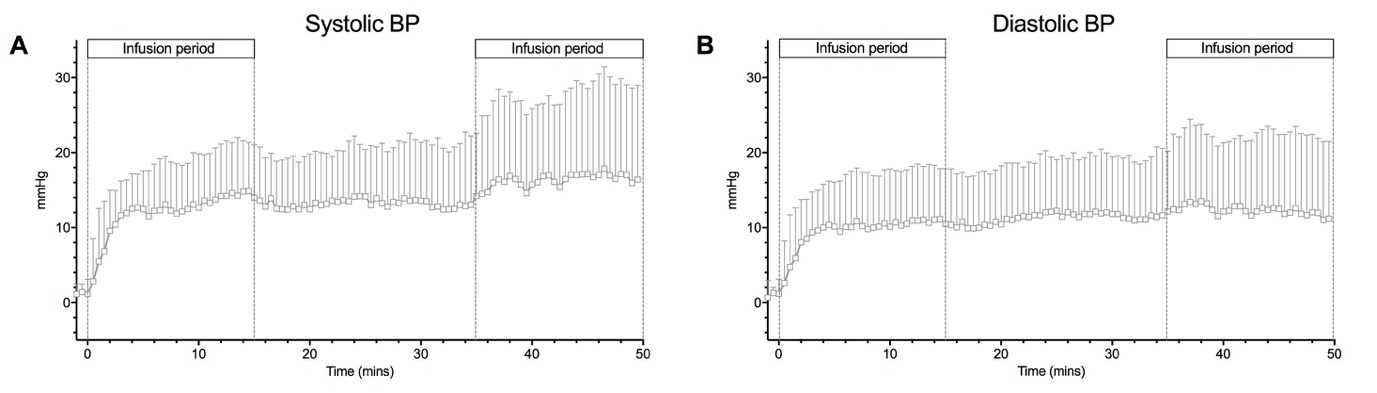


**Figure S2. Renal resistive index and velocity time integral (VTI) with AZ’5538.** C57BL/6J mice were infused with 5% mannitol vehicle followed by AZ’5538. Pulse-wave Doppler ultrasound was used to measure (A) renal blood velocity, (B) VTI and (C) resistive index (A) in the renal artery. All data are mean±SD, n=4 and analysed by two-way ANOVA, n=4. P values shown for vehicle vs drug treatment.

**Figure S3. Heart rate effect of AZ’5538 are GPR81 dependent.** *Gpr81^-/-^* mice (open circles; n=9) and WT littermates (closed squares; n=10) were infused IV with AZ’5538 (1µmol/kg/min) for 15-minutes and heart rate measured from the carotid artery. Data are mean±SD from baseline. Statistical comparisons were made by two-way ANOVA: p=0.026 for the effect of genotype.

**Figure S4. Examples of glomerular arteriole Gpr81 expression by in situ hybridization.** Positive Gpr81 mRNA expression shown by red punctate dots in wildtype mouse glomeruli and adjacent arterioles of the kidney cortex. Scale bars are 50μm.


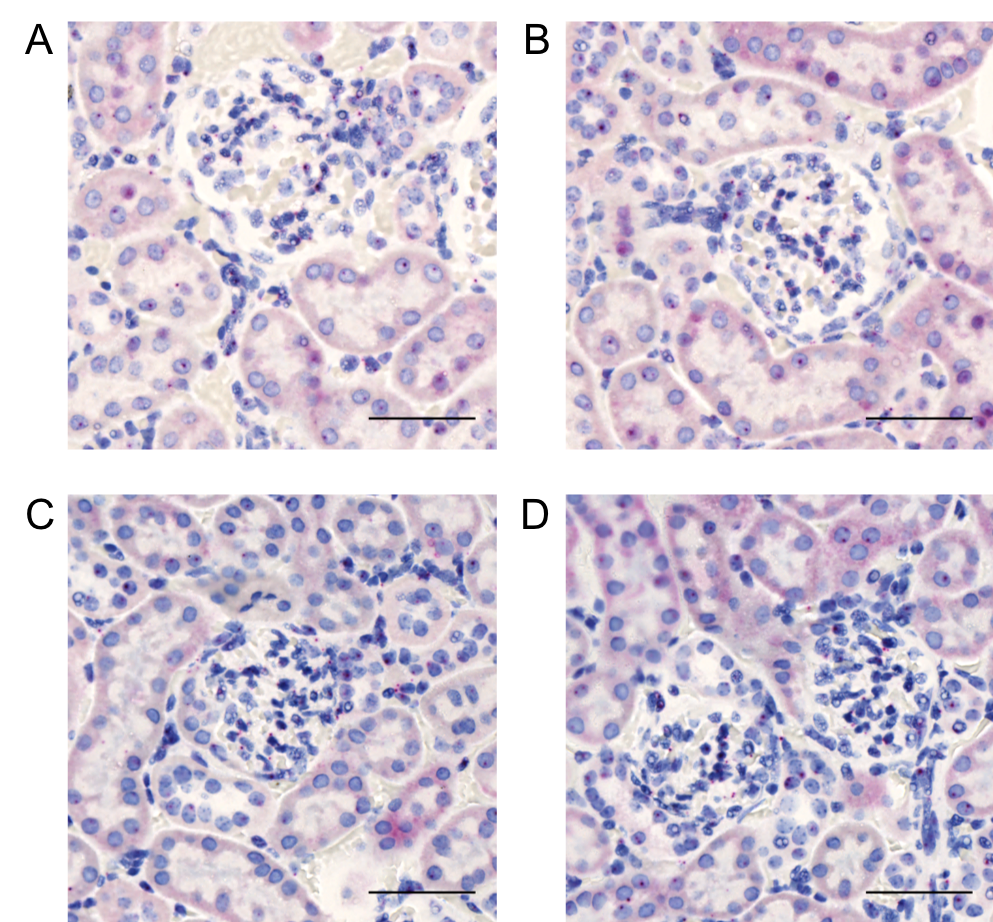


**Figure S5. Arteries from Gpr81^-/-^ mice do not express Gpr81 shown by in situ hybridization.** Positive Gpr81 mRNA expression would be shown by red punctate dots. Aortas (A and B) and renal arteries (C and D) from Gpr81^-/-^ mice do not show any positive staining. Scale bars are 20μm.


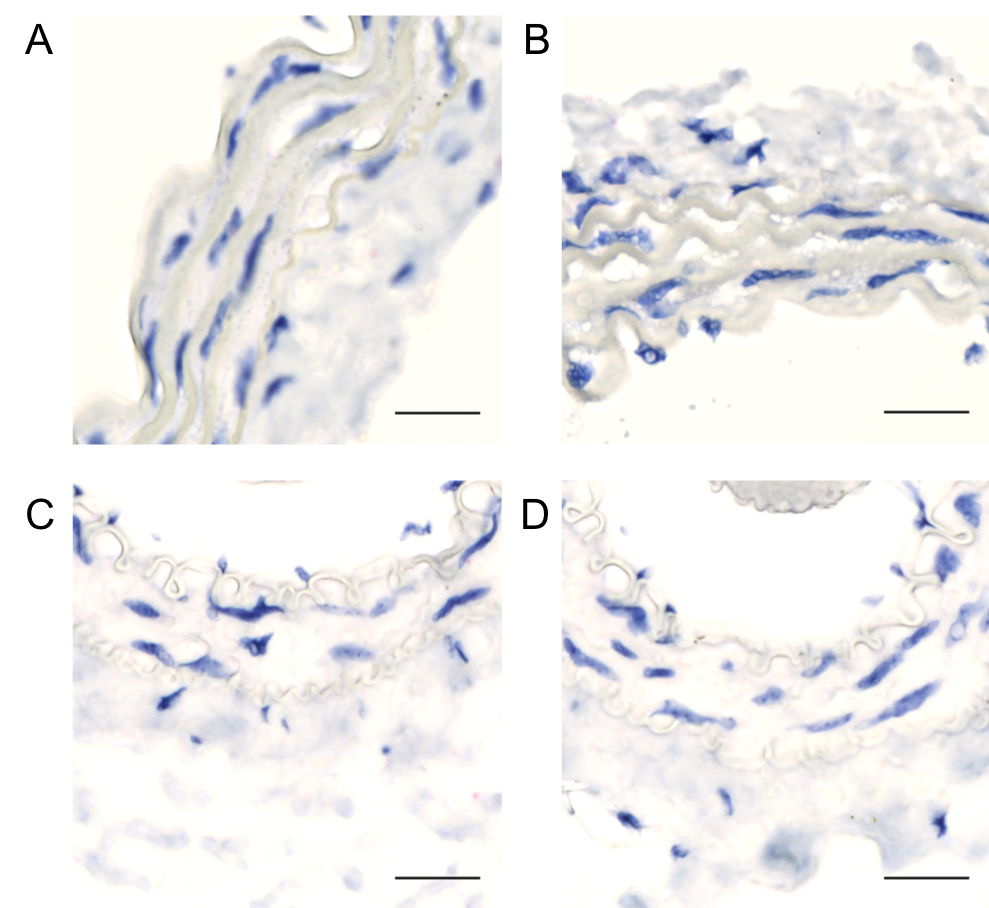


**Figure S6. Role of the endothelin system.** The terminal endothelin-1 concentration in C57Bl/6J mice infused with either vehicle (open square; n=8) or AZ’5538 (1µmol/kg/min black square; n=8) for 15 minutes in (A) whole kidney and (B) aorta homogenates. (C) *Edn1* gene expression in whole kidney homogenates normalised to housekeeper gene *Hprt.* Individual data-points and mean are shown. Data anlysed by unpaired t-test with Welch’s corrections.

**Figure S7. Endothelin receptor antagonists have no effect on baseline BP.** Vehicle and endothelin receptor antagonists were infused intravenously into C57BL/6J mice over a 25 minute period. Systolic (A, C, E) and diastolic (B, D, F) blood pressure was recorded during infusion of Bosentan (ETRA and ETRB mixed antagonist – A and B), BQ123 (ETRA antagonist – C and D) and BQ788 (ETRB antagonist – E and F). All data are mean±SD from baseline, n=4-7. All data was assessed by two-way ANOVA and no significant differences found.

**Figure S8. Effect of endothelin receptor antagonism on GPR81 mediated diastolic blood pressure changes.** Vehicle and two doses of endothelin receptor antagonists were infused intravenously into C57Bl/6J mice over a 25 minute period prior to being infused alongside AZ’5538 for 15 minutes at 1µmol/kg/min. Bosentan (ETRA and ETRB mixed antagonist – A and B), BQ123 (ETRA antagonist – C and D) and BQ788 (ETRB antagonist – E and F) were compared to vehicle. All data are mean±SD from baseline, n=4-7. Diastolic blood pressure (A, C, E) was recorded and analysed by two-way ANOVA while area under the curve data was calculated and analysed by one-way ANOVA.

**Figure S9. Gpr81^-/-^ mice have reduced injury following renal ischemia reperfusion.** Renal ischemia-reperfusion injury or a sham operation was performed on Gpr81^-/-^ (n=4/6) and wild-type mice (n=6/4). One week later, the renal expression of the following genes was measured by qPCR: A) *Adgre1*  (encoding the macrophage marker F4/80); B) *Cxc10* (encoding C-X-C Motif Chemokine Ligand 10 aka interferon-γ inducible protein 10*); C) Ednra* (encoding endothelin receptor A)*;* and D) *Ednrb* (encoding endothelin receptor B). Expression is normalised to housekeepers*;* individual data points and group mean±SD are shown. Statistical comparisons were made by one-way ANOVA with Holm-Sidak test for planned comparisons with p-values as indicated.


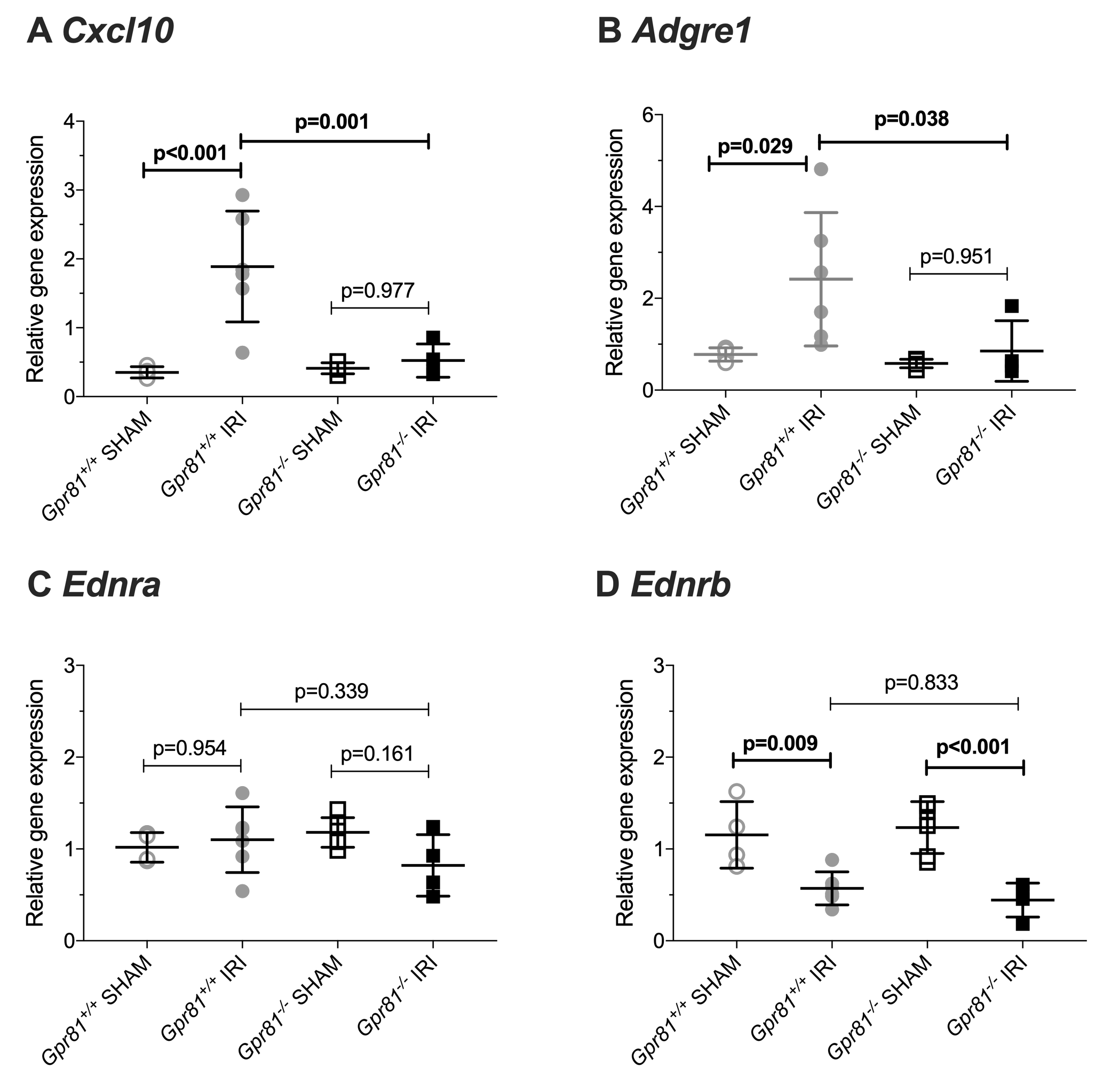

Supplement: Supplementary file 2 [file hyp-75-1213-s002.docx]
