# Supplementary material for: Endothelin-1 Mediates the Systemic and Renal Hemodynamic Effects of GPR81 Activation
Source: Hypertension. 2020 Mar 23;75(5):1213–22. doi: 10.1161/HYPERTENSIONAHA.119.14308 (PMC7176350; doi:10.1161/HYPERTENSIONAHA.119.14308)
Supplement: Supplementary file 3 [file hyp-75-1213-s003.pdf]

*** Short In Vivo Checklist**

AHA - Preclinical Animal Testing: Prevention of bias is important for experimental cardiovascular research. ***This short checklist must be completed, and the answers should be clearly presented in the manuscript as well.*** *The checklist will be used by reviewers and editors but will not be published. If a revision is requested, you will be required to complete at revision submission a more detailed checklist that will be published with the accepted article.*

This study involves animals:
Yes

Animals

Species, age, sex, strains, and sources of animals are described:	Yes

Randomization

Randomization and allocation concealment were performed:	No

Blinding

Blinding was performed:	Yes

Inclusions and Exclusions (a)

Specific criteria for inclusions and exclusions are specified:	Yes

Inclusions and Exclusions (b)

Criteria for inclusions and exclusions were set before the study:	Yes

Reporting of Excluded Animals

All animals excluded after the randomization are reported:	Yes

Statistical Methods

Statistical Methods are described:	Yes

Date completed: 02/18/2020 18:09:19
User pid: 21437
